# Natriuretic Peptide System Activation in Acute Heart Failure Patients with Diabetes

**DOI:** 10.1155/2017/1426705

**Published:** 2017-08-15

**Authors:** Filipe M. Cunha, Joana Pereira, Pedro Marques, Helena Moreira, Pedro Rodrigues, Maria João Pinto, Patrícia Lourenço, Paulo Bettencourt

**Affiliations:** ^1^Serviço de Endocrinologia do Centro Hospitalar de Trás-os-Montes e Alto Douro, Vila Real, Portugal; ^2^Serviço de Medicina Interna do Centro Hospitalar de São João, Porto, Portugal; ^3^Faculdade de Medicina da Universidade do Porto, Porto, Portugal; ^4^Hospital CUF Porto, Porto, Portugal

## Abstract

**Background:**

Elevated B-type natriuretic peptide (BNP) is a hallmark in heart failure (HF). Diabetic patients with chronic HF seem to have higher BNP than nondiabetics. We studied, in acute HF, if BNP levels are different between diabetics and nondiabetics.

**Methods:**

From a prospectively recruited population of acute HF patients, we selected a convenience sample. In pair-matched analysis, each diabetic patient was matched with a nondiabetic of the same age (±1 year), gender, and according to left ventricular systolic dysfunction. Diabetics and nondiabetics were compared. Cox-regression analysis was used to analyse the prognostic impact of diabetes.

**Results:**

We studied 328 patients, mean age: 78 years, 44.5% male. Diabetics were more often hypertensive and had ischemic HF; they had higher body mass index, lower haemoglobin, and worse renal function. Diabetics were more often discharged on ACE inhibitors/ARB, antiplatelet therapy, and statins. Neither admission nor discharge BNP values differed between diabetics and pair-matched nondiabetics. One-year mortality was also nondifferent between pairs of diabetics and nondiabetics: 44 (26.8%) and 46 (28.0%), respectively. HR for 1-year mortality in diabetics was 1.00 (95% CI: 0.82–1.24) compared with nondiabetics.

**Conclusions:**

HF patients with diabetes have similar neurohumoral activation when compared with nondiabetics. One-year mortality is also nondifferent after matching for age, gender, and systolic function.

## 1. Introduction

Diabetes is a known risk factor for incident heart failure (HF) [1, 2] and has also been reported to associate with worse outcome in patients with established HF [3–7].

B-type natriuretic peptide (BNP) is produced by the stressed heart, namely, the stressed ventricles, and is a mostly friendly counter-regulatory peptide [8]. Increased natriuretic peptide levels are, however, insufficient to counteract the activation of other systems like the renin-angiotensin-aldosterone or the sympathetic nervous system and, therefore, water and sodium retention and ventricular remodeling ensue [9]. Natriuretic peptides are useful in the diagnosis of HF and also in risk stratification and prognostic prediction in both the acute and chronic settings [8–10].

Diabetes can contribute to HF development and progression in multiple ways: metabolic and functional alterations, hyperglycemia-induced structural abnormalities, microvascular dysfunction, cardiac autonomic neuropathy, and neurohormonal abnormalities [11–14]. Among patients with no apparent cardiovascular disease, diabetics seem to have higher BNP levels than nondiabetics [15], and in type 2 diabetic patients, BNP levels correlate well with systolic and diastolic left ventricular dysfunction [16]. BNP levels are higher in HF patients with systolic dysfunction when compared to those of patients with HF with preserved ejection fraction [16, 17]. Female gender, older age, and worse renal function are also associated with higher BNP levels [18, 19]. On the other hand, BNP levels are lower in patients with higher body mass index (BMI) [20].

Diabetic patients with chronic advanced HF seem to have higher BNP than their nondiabetic counterparts [21]. In the acute HF setting, difference in neurohumoral activation, as measured by BNP levels, between diabetic and nondiabetic patients was not previously studied.

We aimed to study if the neurohumoral activation is different between diabetic and nondiabetic acute HF patients matched for age, gender, and left ventricular systolic dysfunction.

## 2. Methods

This study is a post hoc analysis conducted in a prospectively recruited population of 657 acute HF patients. From the prospectively recruited population of patients admitted in the Internal Medicine department of Hospital São João between January 2009 and December 2010 due to acute HF as part of an acute HF registry, we retrospectively selected a convenience sample of 328 patients. As part of the registry's protocol, all patients admitted with the primary diagnosis of acute HF were eligible for inclusion in the registry. Both patients with acute de novo and worsening chronic HF were studied. The 2008 European Society of Cardiology guidelines were used for the diagnosis of HF [22]. Patients were excluded if acute coronary syndrome was the cause of acute HF and if symptoms were ultimately attributed to conditions other than HF. Physicians treating acute HF patients admitted during this time period were aware of the ongoing HF registry. The patients' treatment strategy, timing of discharge, and discharge medication were at the discretion of the attending physician.

Also, as part of the registry's protocol, an echocardiogram was performed to all patients during hospitalization and both patients with systolic dysfunction and those with HF with preserved ejection fraction were included. Left ventricular ejection fraction >50% was considered preserved systolic function. Type 2 diabetes mellitus was defined as either a reported history of type 2 diabetes mellitus or the current prescription of either an oral hypoglycaemic drug or insulin. In patients with no known history of type 2 diabetes and a glycated haemoglobin (HbA1c) ≥6.5% measured during hospitalization, a new diagnosis of diabetes was made. BNP nonresponse was defined as an increase or <30% decrease in BNP during hospitalization. BMI was calculated as the body mass (Kg) divided by the square of the body height (m). Demographic characteristics, medications in use upon hospitalization, discharge medication, and comorbidities were recorded. All patients were drawn a venous blood sample within the first 48 h of admission and in the discharge day (registry's protocol). BNP determination is a routine laboratory procedure in our hospital; a chemiluminescent microparticle immunoassay (2-step immunoassay) is performed using an Architect i2000® automated analyzer (Abbott, Lisbon, Portugal). The measurement range of this assay is 10–5000 pg/mL. Serum sodium, creatinine, and C-reactive protein were measured using conventional methods with an Olympus AU5400® automated clinical chemistry analyzer (Beckman-Coulter®, Izasa, Porto, Portugal). Haemoglobin was obtained using an automated blood counter Sysmex® XE-5000 (Emilio de Azevedo Campos, Porto, Portugal). HbA1c was determined by an ion-exchange high-performance liquid chromatography system with a D-10 Bio-Rad analyzer (Bio-Rad, Porto, Portugal).

Patients were followed up for 1 year, and their vital status 1-year postdischarge was ascertained by consulting hospital registries and by telephone contact with the patients or their relatives. We analysed and compared admission and discharge BNP levels in patients with diabetes and in age-, gender-, and systolic function-matched nondiabetic patients.

The registry's protocol conforms to the ethical guidelines of the Declaration of Helsinki, and it was approved by the local ethics committee. Patients provided informed consent.

### 2.1. Statistical Analysis

From the acute HF patients prospectively recruited, we selected a convenience sample to conduct a 1 : 1 pair-matched study. Each diabetic patient was matched with a nondiabetic one of the same age (±1 year), gender, and left ventricular systolic function (reduced or preserved). Patients that were newly diagnosed with type 2 diabetes were not included in the analysis. Patients with primary valvular disease were also not considered in the selection of this convenience sample. Diabetics and nondiabetics were compared. We used a McNemar test to compare categorical variables; a paired samples *t*-test was used for normally distributed continuous variables and a Wilcoxon test for nonnormally distributed continuous variables. A linear regression analysis was used to study the influence of diabetes on admission and discharge BNP levels; adjustments were made considering BMI, atrial fibrillation, and serum creatinine. A Cox-regression analysis was used to analyze the impact of diabetes in 1-year all-cause mortality in this convenience sample of individuals matched in age, gender, and systolic dysfunction.

## 3. Results

From a total of 657 patients hospital admitted with the primary diagnosis of acute HF, 267 (40.6%) had a known diagnosis of type 2 diabetes mellitus and a new diagnosis of type 2 diabetes was made in 63 patients (9.6%).

We analysed 328 acute HF patients: 164 diabetics and 164 nondiabetics. Mean age was 78 (±9) years, 146 (44.5%) were male, 133 (40.5%) had HF of ischemic aetiology, and 212 had reduced ejection fraction. Patients studied had elevated comorbidity burden, and 58.5% were admitted in NYHA class IV. Admission BNP [median (interquartile range)] in the study sample was of 1527.0 (806.0–2557.1) ng/L, and discharge BNP was of 726.0 (279.0–1301.0) ng/L.

Table 1 shows the comparison between pairs of age-, gender-, and systolic dysfunction-matched diabetic and nondiabetic acute HF patients. HF patients with diabetes more often had a history of arterial hypertension and HF of ischemic aetiology. Atrial fibrillation was more common in nondiabetic HF patients. Diabetics also had higher body mass index, lower haemoglobin, lower total cholesterol, and a trend towards worse renal function. Almost 80% of the patients were discharged on beta blockers as well as on angiotensin converting enzyme inhibitor (ACEi) / angiotensinII receptor 1 blocker (ARB). Acute HF patients with diabetes were more often discharged on ACEi/ARB, on antiplatelet therapy, and on statins. Neither admission nor discharge BNP was different between diabetics and nondiabetics of the same age, gender, and with similar systolic dysfunction. In a multivariate linear regression analysis, taking into consideration BMI, atrial fibrillation, and serum creatinine, diabetes remained not predictive of admission and discharge BNP levels (Table 2).

During a 12-month follow-up, 90 patients died (27.4%). One-year mortality was nondifferent between pairs of diabetics and nondiabetics: 44 (26.8%) and 46 (28.0%), respectively, *p* = 0.89. The hazard ratio for 1-year mortality in diabetics was 1.00 (95% CI: 0.82–1.24) when compared with nondiabetics with the same age and gender and similar systolic dysfunction.

## 4. Discussion

In our acute HF population, we report that, contrarily to what has been reported in the general population and in the chronic HF setting in nonmatched groups of patients, BNP levels at admission and at discharge are similar between diabetic and nondiabetic patients when they are 1 : 1 pair-matched considering age, sex, and left ventricular dysfunction.

Age, sex, and systolic function are all factors known to influence and affect BNP levels [16–18]. By matching diabetic and nondiabetic patients for age, gender, and presence of systolic dysfunction, we were able to abrogate those influences on BNP levels in order to better study the effect of diabetes in this peptide level in acute HF. We found that BNP levels are similar between diabetic and nondiabetic patients of the same age and sex and with similar systolic function.

Obesity seems to decrease BNP levels. Several hypotheses have tried to explain this phenomenon. Adipose tissue expresses C-type natriuretic peptide receptor, also known as clearance receptors [23]. In obese patients, higher numbers of these receptors could increase the peripheral removal of BNP thus explaining the lower levels of this hormone. Adipokines produced by the fat tissue have also been suggested to influence natriuretic peptide production/secretion by the heart, leading to lower levels in obesity [20, 24, 25]. In our study population, diabetic patients had higher BMI and this could have influenced the BNP levels and in part explain the nonsignificant trend of diabetic and also more obese patients towards lower admission BNP levels. On the other hand, diabetic patients also had more often ischemic HF, history of atrial fibrillation, lower haemoglobin, and a trend towards worse renal function, all of which were shown to increase BNP levels as well [24]. However, after matching for the probably more important confounding variables (sex, age, and systolic dysfunction), even after adjusting for BMI, atrial fibrillation, and serum creatinine, diabetes remained nonindependently associated neither with admission nor with discharge BNP levels. With our study design, patients were matched for three of the variables that are most recognized to influence the BNP levels, and diabetes was still not a significant determinant of BNP levels after adjustment for BMI, cardiac rhythm, and renal function.

Diabetic patients without known cardiovascular disease were reported to have higher NT-proBNP levels than nondiabetic controls [15]. It has also been reported that diabetic patients with chronic HF had higher BNP and a trend towards higher NT-proBNP levels than their nondiabetic counterparts [21]. Possible hypothesis for these findings was worse diastolic function in diabetic patients [15, 21] or decrease myocardial relaxation as a consequence of altered Ca^2+^ homeostasis due to impaired ATP production in diabetes [15]. Diastolic function was not measured in neither study [15, 21]. In our patient population, we also did not properly assess diastolic function; however, the E/E' ratio was similar between the two groups, making this explanation less likely, at least in acute HF.

In an acute decompensate HF setting, other more strong influences on BNP production like hypoxia or mechanical strain to the heart walls [20] may overwhelm the effect of diabetes. In chronic, more stabilized HF patients, the influence of diabetes could become more noticeable. This could possibly explain the difference between BNP levels in chronic and acute diabetic HF patients. Other hypothesis is that factors such as age, gender, and systolic function are more important than diabetes in determining the natriuretic peptide levels, and once such parameter's influence is abolished, no influence is left over for diabetes in BNP levels. In other words, natriuretic peptide levels in the acute setting are explained by factors other than the coexistence of diabetes.

Also of note is the fact that, once matching for age, sex, and left ventricular systolic function and eventually consequent nondifferent levels of admission and discharge BNP levels between groups of diabetic and nondiabetic patients, a similar association with 1-year mortality was seen in diabetics and nondiabetics. These results suggest that it is possible that the reported association between diabetes and poorer prognosis in HF may be more dependent of the clustering of certain factors or, in other words, a certain environment surrounding diabetic patients than of diabetes per se.

Our study has some limitations that are worth to mention. It is a single-centre study with associated generalizability concerns. Although the whole patient population was prospectively recruited as part of an acute HF registry, we conducted a retrospective analysis on a convenience sample selected from such a registry; retrospective analysis has known inherent shortcomings. Also, the fact that the study is based on a convenience sample, that was necessary for matching purposes, implies that it may not be representative of the whole patient population of acute HF patients seen in everyday clinical practice. It is however a reasonably large nontrial, old, patient population that was followed for a relatively long time. With this matched study design, we were able to abolish the effect of age, gender, and systolic function on BNP levels. Echocardiographic parameters to properly assess diastolic function were not available. Another important setback is the fact that the duration of type 2 diabetes was not known and not accounted for in the analysis; we have, however, excluded patients with newly diagnosed and not previously treated diabetes therefore not including those with shorter disease duration. Apart all the limitations noted, this is, as far as we are aware, the first study to directly compare BNP levels between diabetic and nondiabetic acute HF patients after matching for age, gender, and systolic function.

## 5. Conclusions

HF patients with diabetes have similar admission and discharge BNP levels when compared with nondiabetics. One-year mortality is also nondifferent between diabetics and nondiabetics of the same age and gender and similar systolic function.

## Figures and Tables

**Table 1:** Patients' demographic, clinical, and laboratory characteristics; comparison between diabetic and nondiabetic patients.

	Nondiabetics (*n* = 164)	Diabetics (*n* = 164)	*p* value
*Clinical characteristics*			
Age (years), mean (SD)	78 (9)	78 (8)	0.55
Male sex, *n* (%)	73 (44.5)	73 (44.5)	1.00
LVSD, *n* (%)	106 (64.6)	106 (64.6)	1.00
Arterial hypertension history, *n* (%)	111 (67.7)	145 (88.4)	<0.001
Atrial fibrillation, *n* (%)	85 (51.8)	65 (39.6)	0.04
Ischemic etiology, *n* (%)	56 (34.1)	77 (47.0)	0.02
NYHA class (IV versus others), *n* (%)	97 (59.1)	95 (57.9)	0.91
Heart rate (bpm), mean (SD)	90 (25)	88 (22)	0.48
Systolic blood pressure (mmHg), mean (SD)	133 (31)	137 (28)	0.24
BMI (Kg/m^2^), mean (SD)	25.1 (4.3)	26.6 (4.6)	0.005
*Laboratory parameters at admission*			
Haemoglobin (g/dL), mean (SD)	12.0 (2.2)	11.4 (2.2)	0.02
Sodium (mEq/L), mean (SD)	137 (4)	137 (5)	0.94
Urea (mg/dL), mean (SD)	74 (40)	83 (46)	0.07
Creatinine (mg/dL), mean (SD)	1.50 (0.63)	1.64 (0.68)	0.06
C-reactive protein (mg/L), median (IQR)	21.1 (10.2–63.6)	19.0 (9.9–61.3)	0.73
Total cholesterol (mg/dL), mean (SD)	164 (45)	147 (41)	0.002
BNP (ng/L), median (IQR)	**1664.1 (905.7–2635.7)**	**1394 (773.3–2456.8)**	**0.10**
Discharged BNP (ng/L), median (IQR)	**753.0 (280.7–1237.6)**	**688.0 (275.0–1335.2)**	**0.51**
Nonresponse, *n* (%)	40 (24.4)	50 (30.5)	0.44
*Echocardiographic parameters*			
LV mass (g), mean (SD)	236.9 (84.8)	231.1 (51.2)	0.44
Left atrium size (mm), mean (SD)	4.9 (0.8)	4.7 (0.5)	0.14
LV end-diastolic diameter (mm), mean (SD)	5.7 (1.0)	5.6 (0.8)	0.14
E/E', median (IQR)	16 (12–21)	19 (13–23)	0.27
*Discharge medication*			
ACEi or ARB, *n* (%)	120 (76.2)	142 (86.6)	0.004
Beta blocker, *n* (%)	125 (76.2)	129 (78.7)	0.69
MRA, *n* (%)	43 (26.2)	30 (18.3)	0.09
Loop diuretics, *n* (%)	151 (92.1)	157 (95.7)	0.45
Furosemide dose (mg/day), median (IQR)	80 (40–100)	80 (60–120)	0.02
Statins, *n* (%)	90 (60.4)	117 (71.3)	0.03
Antiplatelet therapy, *n* (%)	100 (61.0)	124 (75.6)	0.01
Mortality at 1 year, *n* (%)	44 (26.8)	46 (28.0)	0.89

ACEi: angiotensin converting enzyme inhibitor; ARB: angiotensin II receptor 1 blocker; BMI: body mass index; BNP: B-type natriuretic peptide; IQR: interquartile range; LV: left ventricle; LVSD: left ventricular systolic dysfunction; MRA: mineralocorticoid receptor antagonist; NYHA: New York Heart Association.

**Table 2:** Influence of diabetes on admission and discharge BNP levels: linear regression analysis.

	Admission BNP		Discharge BNP	
	*β* coefficient (95% CI)	*p* value	*β* coefficient (95% CI)	*p* value
Diabetes mellitus	−116.5 (−347.0; 114.0)	0.32	−103.2 (−302.2; 95.8)	0.31

Adjusted for body mass index, admission creatinine, and atrial fibrillation. BNP: B-type natriuretic peptide.
